# Chemical Examination of Some Substances Used in Ceylon as Remedies against the Bites of Venomous Serpents

**Published:** 1818-04

**Authors:** John Davy


### For the London Medical and Physical Journal.

*Chemical Examination of some Substances used in Ceylon as Remedies against the Bites of venomous Serpents.* By JOHN DAVY, M.D. F.R.S. Communicated by Sir H. DAVY.

IT is well known that certain substances or preparations are used in India by the native empirics, or snake-charmers, for curing the bites of venomous snakes. As some confidence has been placed in these nostrums, not only by the Indians but by some of the European settlers, it was interesting to ascertain whether any real virtue belonged to them. This object has been undertaken by Dr. Davy, who at present resides in Ceylon; and he has communicated the result of his researches (of which an abstract is here given) to the Asiatic Society of Calcutta.

The snake-stones, as they are called, which Dr. Davy examined, were of three kinds:—One, by minute analysis, was found to be merely calcined bone; another was carbonate of lime, coloured by vegetable matter; the third was a bezoar stone. Of these, the two first had some adhesive powers when applied to the tongue; but the last had no such power.

Dr. Davy decides, that these stones are of no use whatever as applied to the wounds produced by the bites of serpents; and he refers the pretended cures produced by them to Nature, or to the circumstance that they have been applied to wounds produced by snakes which are not venomous.

Of eleven different species of snakes which the author has examined, all of which were believed by the natives to be poisonous, Dr. Davy found only three to be really so; and the bites of two of these (the *Cobra di capello* and the *Polonga*) only are mortal, and that under very peculiar circumstances.

Dr. Davy concludes by stating, that the sooner the belief in snake-stones is exploded the better, as much time is lost in applying imaginary remedies, and some lives are lost which might have been saved, and much suffering occasioned which might have been prevented, by efficient means of cure.—*Phil. Mag.*

